# Calanquinone A suppresses glioma progression via STAT3-mediated regulation of c-Myc and MMP9

**DOI:** 10.1007/s12672-025-03279-4

**Published:** 2025-08-04

**Authors:** Wen-Chi Hsieh, Chun-Yu Lin, Hsuan‐Cheng Wu, Huang-Wei Lo, Chiung-Yuan Ko, Jian-Ying Chuang, Tsung-I. Hsu, Tsui-Hwa Tseng, Teng-Wei Huang, Shao-Ming Wang

**Affiliations:** 1 Neuroscience and Brain Disease Center, China Medical University, Taichung, Taiwan; 2 Graduate Institute of Biomedical Sciences, College of Medicine, China Medical University, Taichung, Taiwan; 3 School of Medicine, College of Medicine, China Medical University, Taichung, Taiwan; 4 School of Medicine, College of Medicine, National Sun Yat-sen University, Kaohsiung, Taiwan; 5 Ph.D. Program in Medical Neuroscience, College of Medical Science and Technology, Taipei Medical University, Taipei, 110301 Taiwan; 6 International Master Program in Medical Neuroscience, College of Medical Science and Technology, Taipei Medical University, Taipei, 110301 Taiwan; 7 Department of Medical Applied Chemistry, Chung Shan Medical University, Taichung, 402306 Taiwan; 8 Ph.D. Program for Aging, China Medical University, Taichung, Taiwan

**Keywords:** Calanthe arisanensis, Calanquinone a, Phytochemical, Glioblastoma multiforme, STAT3, Migration

## Abstract

Calanthe arisanensis, a traditional medicinal orchid, contains Calanquinone A, a quinone-type compound with reported anticancer properties. Glioblastoma multiforme (GBM) is a highly aggressive brain tumor with limited treatment options and a poor prognosis. This study investigates the molecular mechanisms underlying the antiproliferative and antimigratory effects of Calanquinone A in GBM. Human GBM cell lines (U87MG) and patient-derived primary GBM cells (Pt#3) were treated with Calanquinone A (1–2.5 µM), and effects on cell viability, proliferation, and migration were assessed using CCK-8, trypan blue exclusion, colony formation, wound healing, transwell migration, and F-actin staining assays. Calanquinone A significantly reduced GBM cell viability and migration. Western blot analysis revealed that Calanquinone A inhibited STAT3 phosphorylation at Tyr705, leading to downregulation of its downstream targets c-Myc and MMP9. STAT3 overexpression reversed these effects, confirming the involvement of STAT3-dependent pathways. In a mouse xenograft model, Calanquinone A suppressed tumor formation in vivo. Molecular docking analysis further revealed a potential direct interaction between Calanquinone A and STAT3, supporting a direct mechanism of action. These findings demonstrate that Calanquinone A exerts potent antitumor effects in glioblastoma by targeting the STAT3/c-Myc and STAT3/MMP9 signaling axes, supporting its potential as a natural STAT3-targeting agent for GBM therapy.

**Graphical abstract:**

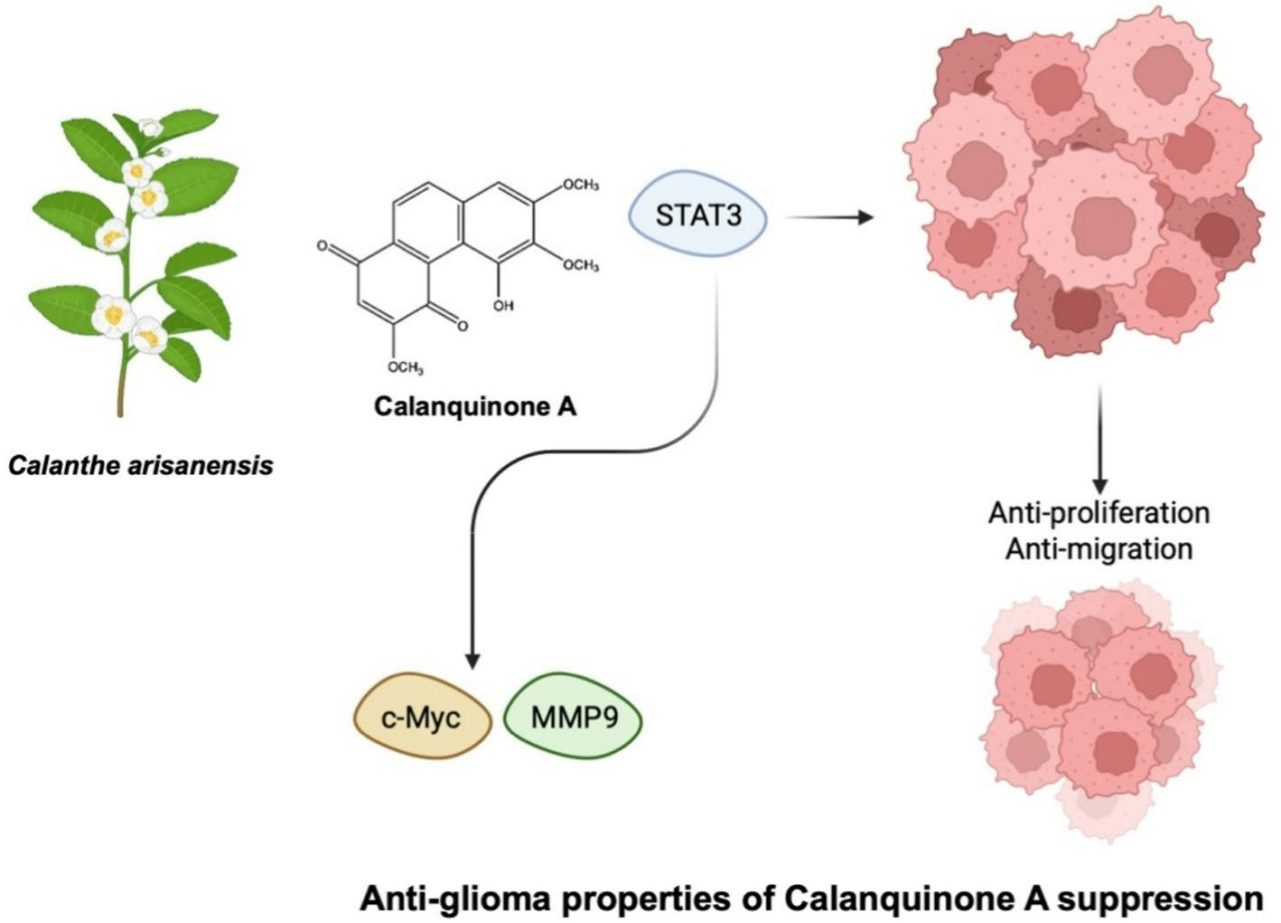

**Supplementary Information:**

The online version contains supplementary material available at 10.1007/s12672-025-03279-4.

## Introduction

Glioblastoma (GBM) is a grade IV astrocytic glioma that is the most malignant brain tumor, according to the World Health Organization (WHO) classification of brain tumors. It aggressively invades the surrounding brain regions, leading to death [1]. Currently, the standard therapy for GBM is safe surgical resection, followed by radiotherapy (RT) or radiochemotherapy combined with temozolomide (TMZ). Other adjuvant chemotherapies may also be used [2]. Despite these treatments, the overall survival rate remains at < 10% [2]. Therefore, there is an urgent need to identify novel candidate compounds for the treatment of GBM.

Several pathways participate in the development and progression of GBM, including the receptor tyrosine kinase (RTK), TP53, retinoblastoma protein (RB), and JAK/STAT pathways [3–6]. Among these, JAK/STAT has been identified as a key signaling pathway associated with replication, apoptosis, migration, invasion, and immunosuppressive functions in tumor pathogenesis. Among the STAT family, STAT3 exhibits the most extensive oncogenic activity in GBM, with up to 60% of human GBM cases positive for p-STAT3 expression [4, 7]. STAT3 normally resides in the cytoplasm. Upon phosphorylation at tyrosine-705 (Y705), STAT3 forms a homodimer and translocates into the nucleus to regulate the transcription of genes such as c-Myc, Bcl-2, Cyclin D1, and MMP9. These downstream factors promote the survival, proliferation, and metastasis of GBM [7, 8]. Furthermore, nuclear factor-κB (NF-κB) is activated in GBM [9]. Among its five members, namely p50, p52, p65 (Rel A), Rel B, and c-Rel, classic NF-κB comprises the p50-p65 heterodimer, which enters the nucleus to regulate gene expression [10]. Furthermore, dysregulation of NF-κB signaling leads to glioma cell survival, chemoresistance, and proliferation [11, 12].

Calanquinone A is a chemical compound isolated from *Calanthe arisanesis* [13]. Traditionally, Calanthe arisanensis has been used in East Asian herbal decoctions and medicinal teas for its ethnopharmacological properties, including applications in inflammation and traumatic injuries [14]. This raises the potential for its bioactive constituents to be developed as dietary supplement ingredients. Calanquinone A has been shown to reduce glioblastoma multiforme (GBM) cell survival by depleting glutathione levels and inducing DNA damage [13]. Additionally, it is not a substrate of P-glycoproteins [13]. Despite these findings, the effect of Calanquinone A on cell proliferation and migration remains poorly understood. Therefore, the aim of this study was to investigate the impact of Calanquinone A on these cellular processes and to elucidate the underlying molecular mechanisms involved.

Our results demonstrate that Calanquinone A reduces glioma cell survival and proliferation by downregulating c-Myc expression. In addition, Calanquinone A inhibits cell migration through the upregulation of E-cadherin and the downregulation of MMP9. Mechanistically, we identified STAT3, rather than p65, as the key transcription factor mediating these effects. Furthermore, Calanquinone A suppresses tumor formation in vivo, likely through direct interaction with STAT3. Taken together, by elucidating the role of Calanquinone A in GBM cell proliferation and migration and its underlying mechanisms, our findings support its potential application as a novel anti-GBM agent.

## Materials and methods

### Reagents

Calanquinone A was synthesized, and its structural identification and purity were reported by Yean-Jang Lee [13]. We sincerely thank Tsui-Hwa Tseng, Yean-Jang Lee, and their team for their generous support. It was dissolved in dimethyl sulfoxide (DMSO) at a concentration of 5 mM and stored at − 20 °C.

### Cell culture

The human GBM cell line U87MG was obtained from the American Type Culture Collection (ATCC, Manassas, VA, USA), G261 cells were obtained from Cytion (Deutschland), and Pt#3 (patient-derived glioma) GBM cells were generously provided by Dr. Jian-Ying Chuang of Taipei Medical University [15]. All cells were incubated, maintained, and tested in Dulbecco’s modified Eagle’s medium (DMEM, GIBCO, Waltham, MA, USA) supplemented with 10% fetal bovine serum (FBS, CORNING, REF 35-010-CV, Glendale, AZ, USA) and 1% penicillin/streptomycin (GIBCO, Waltham, MA, USA). The HA-STAT3 expression and HA control plasmids were obtained from SinoBiological (Houston, TX, USA). GBM cells were transfected with plasmids using PolyJet reagent (SignaGen Laboratories, Gaithersburg, MD, USA). The transfection reagent was mixed with the plasmid in a 2:1 ratio and incubated for 20 min at room temperature in serum-free DMEM (200 µL). The mixture was subsequently added to the cells and incubated at 37 °C in a 5% CO_2_ incubator for 24 h. All control treatments in the experiments contained 0.1% DMSO.

### CCK8 assay

At 80% confluence, cells were seeded in 24-well plates and incubated for 24 h. The cells were treated with various concentrations of Calanquinone A and incubated for an additional 24 h. Subsequently, 10-fold diluted CCK8 reagent in DMEM was added to the cells, which were subsequently incubated at 37 °C in a 5% CO_2_ incubator for 1 h. Optical density (OD) was measured at 450 nm using a SpectraMax iD3 reader.

### Trypan blue exclusion assay

Cells (1 × 10^5^) were seeded in 6-cm dishes and incubated at 37 °C in a 5% CO_2_ incubator for 16 h. The cells were treated with various doses of Calanquinone A for 24 and 48 h. Following treatment, the cells were trypsinized, and live cells were counted using the trypan blue dye exclusion method with a Luna™ Automated Cell Counter.

### Foci assay

Cells (5 × 10^3^) were seeded in 6-cm dishes for 16 h. The cells were treated with various doses of Calanquinone A for 24 h. Following treatment, the medium was replaced, and the cells were incubated for 7 days. Subsequently, cell colonies were stained with 0.05% crystal violet for 10 h. The stained colonies were analyzed using AzureSpot Pro software (Dublin, CA, USA).

### Transwell assay

Briefly, glioma cells (1 × 10^5^ cells/200 µL serum-free DMEM) were seeded in the upper compartment of a Transwell chamber. Following this, 600 µL of 10% FBS DMEM was added to the lower compartment of the Transwell chamber. After 18 h, the cells were removed from the upper membrane, and the lower membrane was stained with 4′,6-diamidino-2-phenylindole (DAPI) for 10 min. The DAPI-positive cells were counted under a microscope.

### Wound healing assay

Glioma cells were seeded in 6-cm dishes and scratched with a culture insert (Ibidi GmbH, Martinsried, Germany). A 90% confluent monolayer of glioma cells was cultured in 10% FBS DMEM, with or without 1 µM Calanquinone A, for 18 h. The scratched area was photographed at 0 and 18 h, followed by quantitative analysis using the NIH ImageJ image processing software.

### F-actin staining

The cells were seeded in a 4-well glass slide (Millicell^®^ EZ SLIDES, Darmstadt, Germany). After 16 h, the cells were treated with 1 µM Calanquinone A, or left untreated, for 18 h. Subsequently, the cells were fixed and permeabilized with 0.1% Triton X-100 in PBS, followed by staining with Alexa Fluor 488 Phalloidin (A12379, Invitrogen™) for 1 h and then DAPI solution for 10 min. Images were captured by confocal microscopy.

### Western blot analysis

Cell lysates from U87MG and Pt#3 cells were treated with or without Calanquinone A and extracted in IP buffer (50 mM NaCl, 0.5% NP-40, and 10 mM Tris-HCl; pH 8.0) containing 1× protease inhibitor at 4 °C for 30 min. Proteins were separated using sodium dodecyl sulfate-polyacrylamide gel electrophoresis (SDS-PAGE) gels and transferred onto polyvinylidene difluoride (PVDF) membranes. The membrane was blocked using a 5% Blotting-Grade Blocker (Catalog no.: #1706404, BIO-RAD) and incubated with the indicated antibodies overnight at 4 °C. After three washes, the membranes were incubated with secondary antibodies at 23 °C for 1 h. After three additional washes with TBST, the protein bands were analyzed using an Azure 400 system. Band intensity and quantification were analyzed using Image Studio Lite (Li-Cor 5.2) in accordance with the manufacturer’s instructions. Notably, the blots were cut prior to antibody hybridization to enable probing with multiple antibodies on different regions of the membrane. Consequently, full-length images of intact membranes are not available.

### Xenograft model

All animal experiments were conducted in accordance with the guidelines and ethical standards approved by the Institutional Animal Care and Use Committee (IACUC) of China Medical University and the Guide for the Care and Use of Laboratory Animals (Protocol No. CMUIACUC-2024-022 and -2022-372). According to IACUC regulations, the maximum permitted tumor diameter is 20 mm. In this study, the largest tumor length observed was 14 mm, which remained within the approved limit. Six-week-old male C57BL/6JNarl mice were obtained from the National Center for Biomodels (NCB), NIAR, Taiwan, and housed in a pathogen-free facility under a 12-hour light/dark cycle with ad libitum access to standard rodent chow and water. G261 glioma cells (3 × 10⁶ cells in 100 µL of a 1:1 mixture of Matrigel and PBS) were subcutaneously injected into the right hind flank. One week after inoculation, mice received intraperitoneal injections of either 100 µL of 2% DMSO in PBS or Calanquinone A (10 mg/kg in 100 µL), three times per week (*n* = 6 per group). Tumor volumes were measured every three days using calipers and calculated with the formula: length × width² × 0.5. After four weeks of treatment, mice were humanely euthanized for further analysis.

### Molecular docking analysis

The 3D structure of Calanquinone A was obtained from the PubChem database (Compound CID: 25195268), and the protein structure of STAT3 was retrieved from the RCSB Protein Data Bank (PDB ID: 6NUQ). Molecular docking was performed using AutoDockTools, and the results were visualized with PyMOL.

### Statistical analysis

Data were collected from a minimum of three independent experiments and analyzed using the Prism software (version 10). Results are expressed as the mean ± standard error of the mean (SEM). Statistical significance was assessed using either a paired Student’s t test, an unpaired Student’s t test, a one-way ANOVA, or a two-way ANOVA, with statistical significance set at *P < 0.05, **P < 0.01, ***P < 0.001, and ****P < 0.0001.

## Results

### Calanquinone A inhibited GBM proliferation

**Fig. 1:**
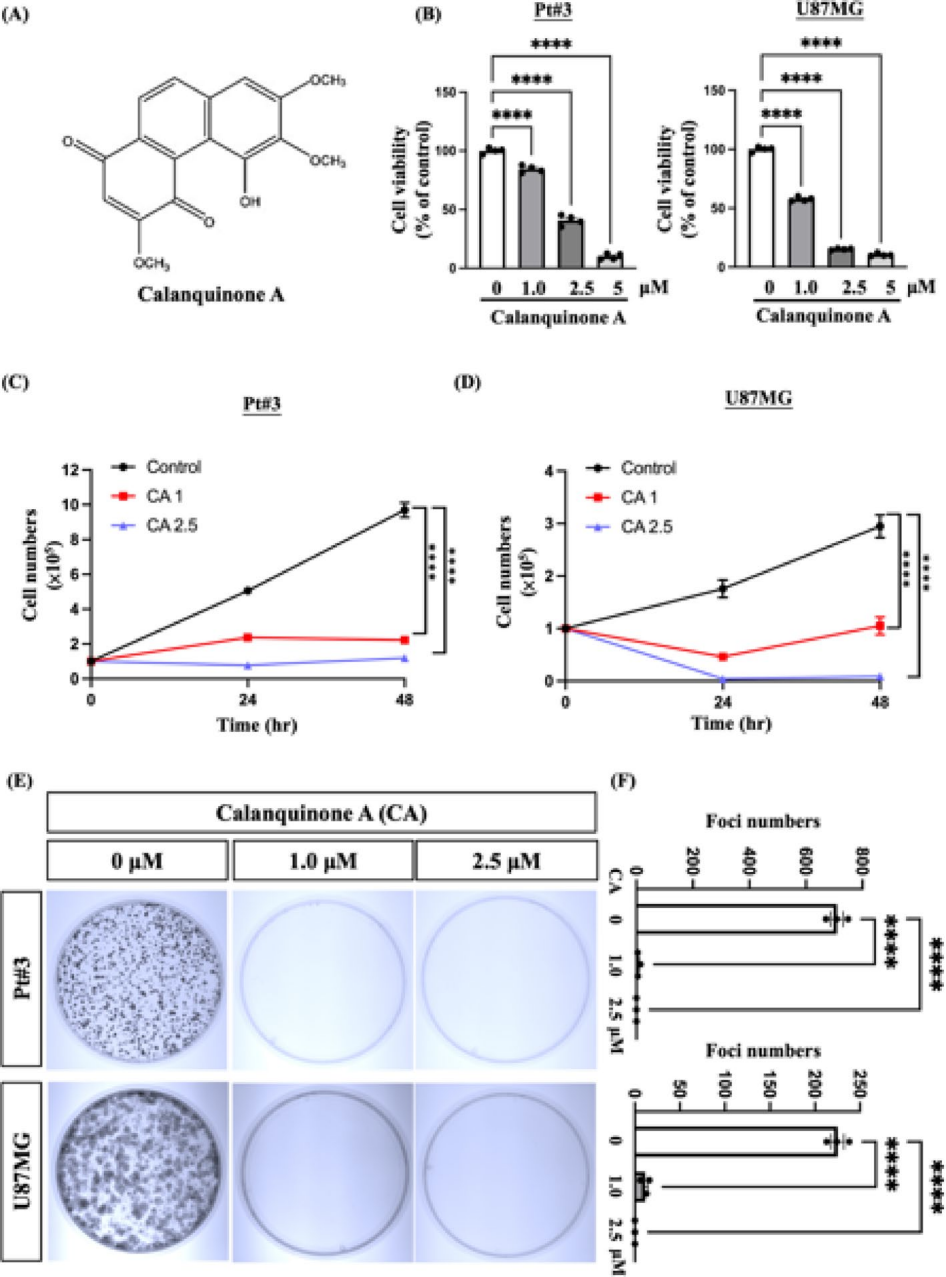
Effect of Calanquinone A on proliferation in glioma. **A** Chemical structure of Calanquinone A, a quinone-type phytochemical isolated from Calanthe arisanensis. **B** Calanquinone A treatment for 24 h reduced cell viability in Pt#3 and U87MG cells, as determined by the CCK8 assay. Data were analyzed using one-way ANOVA with Prism software. ****P < 0.0001, *N* = 4. A trypan blue exclusion assay was used to evaluate short-term proliferation in **C** Pt#3 and **D** U87MG cells after 24 and 48 h of Calanquinone A treatment. **E** A foci assay was performed to evaluate long-term proliferation. Cells were treated with Calanquinone A for 24 h, followed by media replacement and incubation for 14 days. **F** Statistical analysis of results from **E**. Data were analyzed using one-way ANOVA with Prism software. ****P < 0.0001, *N* = 3

Calanquinone A (whose structure is shown in Fig. 1A) significantly reduced the viability of both Pt#3 and U87MG cells, as demonstrated by the CCK8 assay (Fig. 1B). The trypan blue exclusion assay was used to evaluate short-term cell proliferation after treatment with or without Calanquinone A for 24–48 h. The results revealed that 1 and 2.5 µM Calanquinone A inhibited glioma proliferation (Fig. 1C, D). Similarly, the foci assay was used to assess long-term proliferation, revealing that 1 µM Calanquinone A markedly reduced foci formation in Pt#3 and U87MG cells (Fig. 1E, F). Collectively, these data suggested that Calanquinone A inhibited glioma cell proliferation in patient-derived glioma cells and cell lines.

### Calanquinone A reduced glioma cell migration

**Fig. 2:**
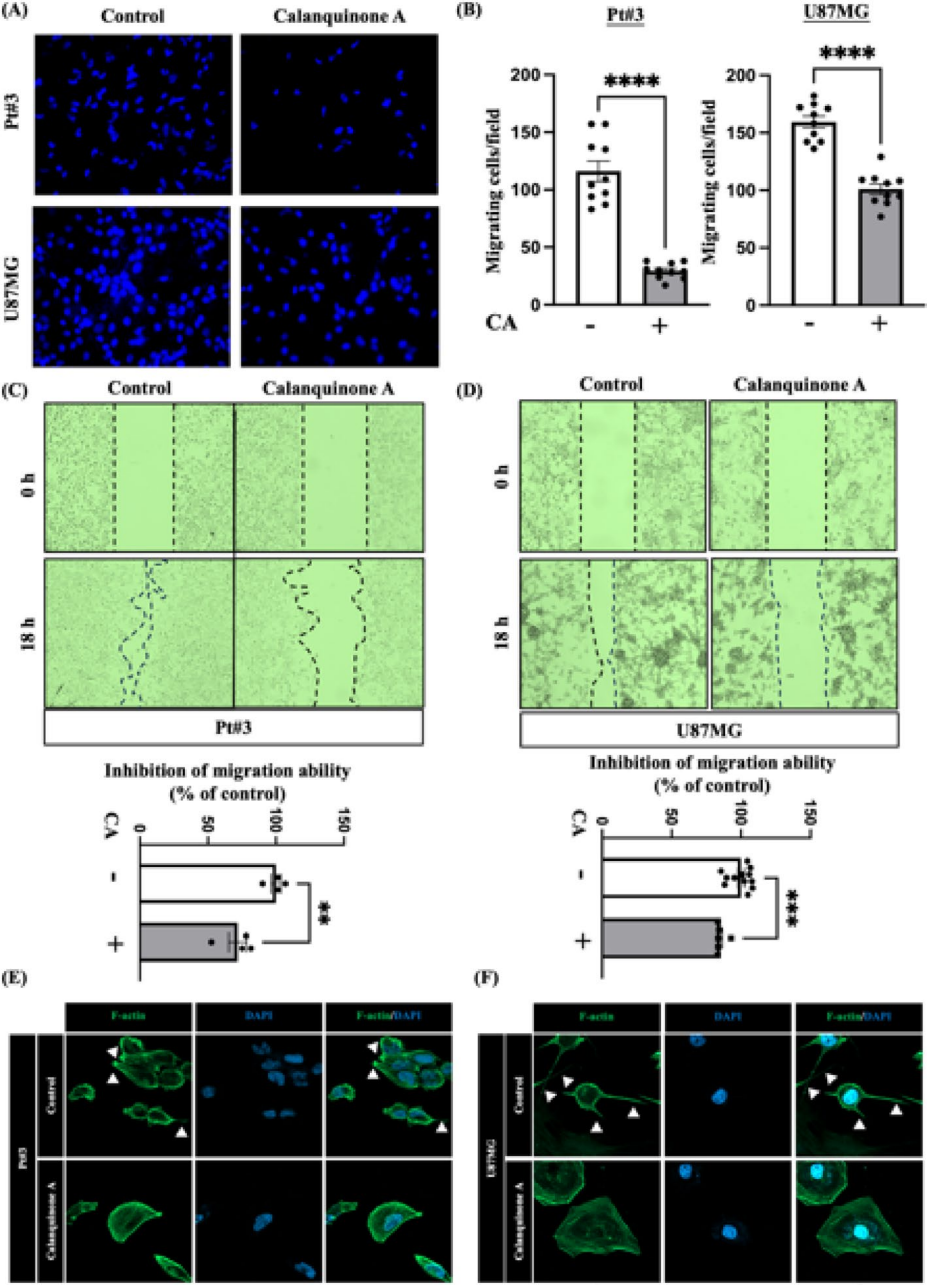
Calanquinone A inhibited glioma cell migration. **A** Calanquinone A reduced the migration of Pt#3 and U87MG cells, as determined using the transwell assay. **B** Statistical analysis of the results shown in **A**. Data are presented as the mean ± SEM; two-tailed unpaired Student’s t test; ****P < 0.0001. **C** Pt#3 and **D** U87MG cells were scratched using a culture insert and treated with or without Calanquinone A. Microphotographs were captured at the time of wound creation (0 h) and after 18 h. The bottom panels show the quantification of the wound closure. Data are presented as the mean ± SEM; two-tailed unpaired Student’s t test; **P < 0.01 and ***P < 0.001. Calanquinone A treatment reduced lamellipodium protrusions in **E** Pt#3 and **F** U87MG cells. White arrows indicate lamellipodium protrusion structures

We investigated whether Calanquinone A influenced cell migration using transwell and wound healing assays. As shown in Fig. 2A and B, Calanquinone A treatment decreased the migration of Pt#3 and U87MG cells after 18 h. Similarly, the wound healing assay demonstrated that the migratory ability of Pt#3 and U87MG cells was reduced after 18 h of Calanquinone A treatment (Fig. 2C, D). Dynamic changes in the cytoskeleton have been observed in the morphological and physical behavior of migrating cells [16]. Among these changes, the lamellipodium actin network facilitates cell migration through actin polymerization [17, 18]. Here, we found that treatment of Pt #3 and U87MG cells with Calanquinone A resulted in reduced lamellipodium protrusions compared to the control groups (Fig. 2E, F).

### Calanquinone A reduced cell proliferation by suppressing c-Myc expression

**Fig. 3:**
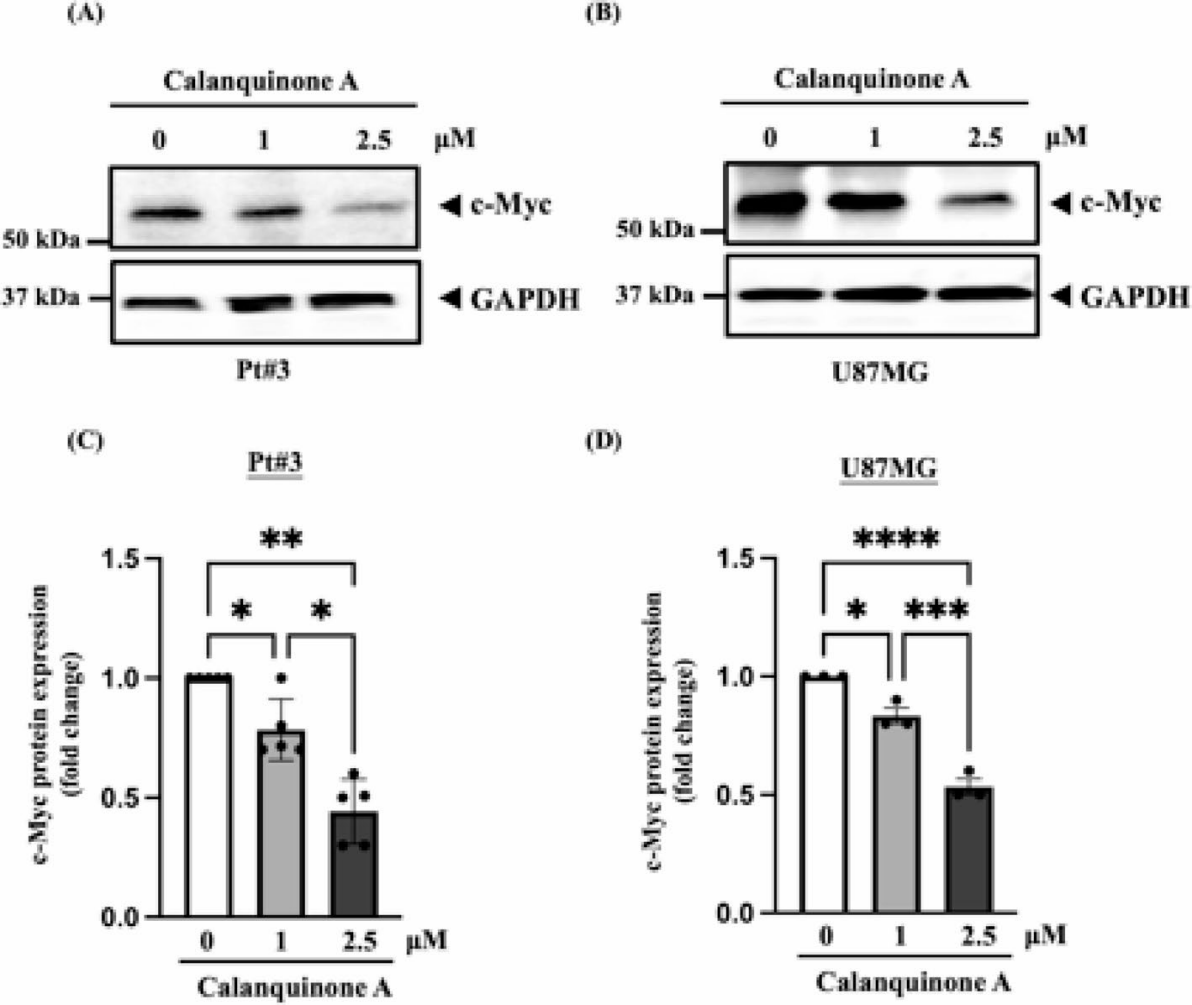
Calanquinone A treatment decreased c-Myc expression in glioma cells. Calanquinone A treatment reduced c-Myc protein expression in **A** Pt#3 and **B** U87MG cells, as determined by western blot analysis. **C** and **D** Quantification of the data from **A** and **B** revealed a decrease in c-Myc expression levels in Pt#3 and U87MG cells following treatment with various doses of Calanquinone A. Data were analyzed using one-way ANOVA with Prism software. *P < 0.05, **P < 0.01, ***P < 0.001, and ****P < 0.0001

Previous studies have demonstrated that c-Myc contributes to glioma cell proliferation and that its inhibition can reduce glioma proliferation [15, 19–21]. Therefore, we investigated whether Calanquinone A inhibits c-Myc expression to suppress cell proliferation. We found that c-Myc expression decreased in Pt#3 and U87MG cells following 24 h of Calanquinone A treatment (Fig. 3A–D). These data suggested that Calanquinone A reduced glioma cell proliferation by downregulating c-Myc expression.

### Calanquinone A influences cell migration by decreasing MMP9 expression

**Fig. 4:**
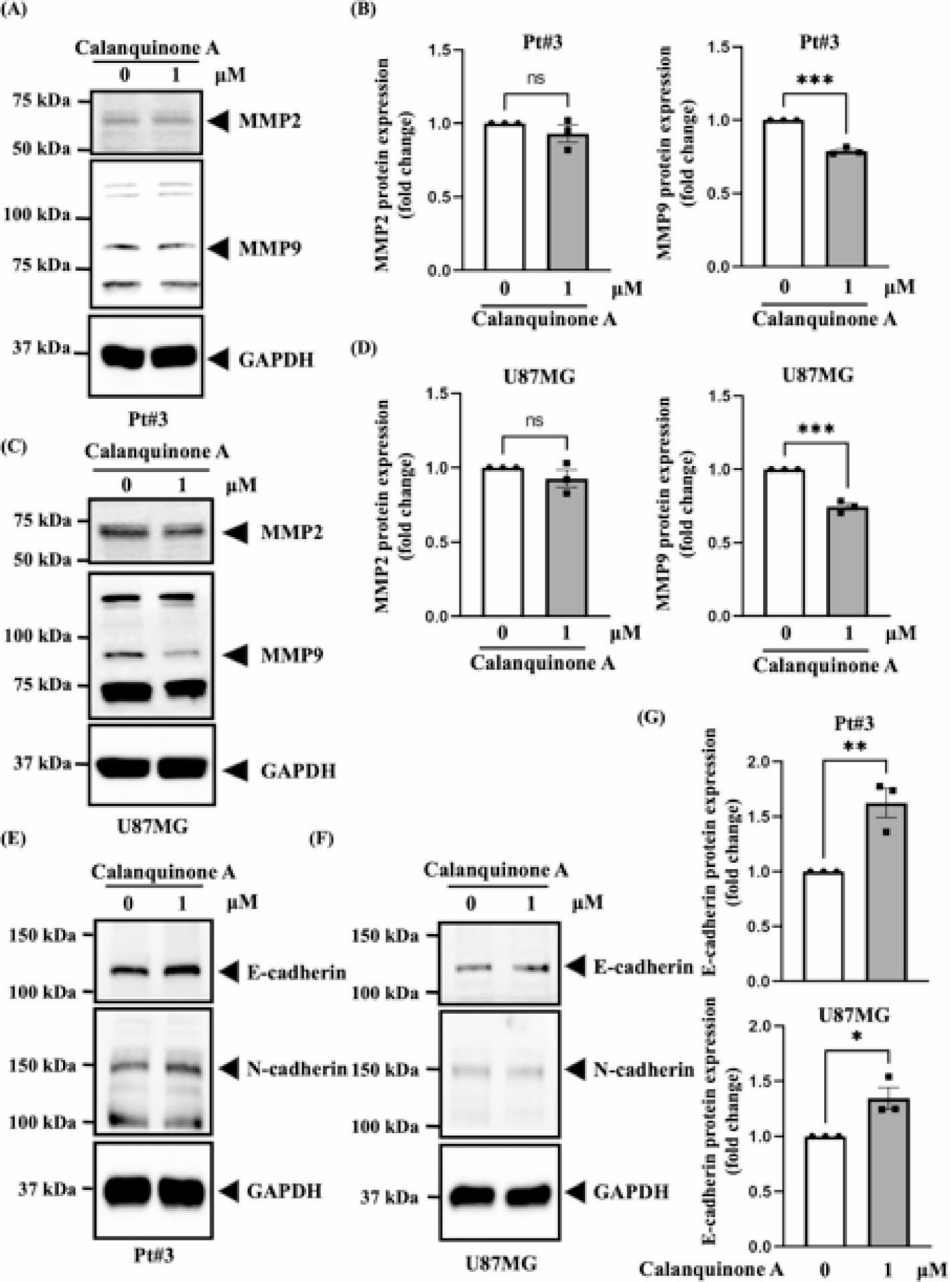
Calanquinone A treatment reduced MMP9 protein expression but increased E-cadherin protein expression in Pt#3 and U87MG cells. **A** Western blot analysis revealed a decrease in MMP9 protein expression in Pt#3 cells following Calanquinone A treatment, but no significant change in MMP2 expression. **B** Quantification of MMP2 and MMP9 levels from **A** reveals a reduction in MMP9 expression. **C** MMP9 expression was decreased in U87MG cells, while MMP2 expression remained unchanged. **D** Quantification of data from **C** further confirms the reduction in MMP9 expression. Data are expressed as the mean ± SEM; two-tailed unpaired Student’s t test; ***P < 0.001. **E** Calanquinone A treatment increased E-cadherin protein expression in Pt#3 and **F** U87MG cells. **G** Statistical analysis of **E** and **F** confirmed the increased E-cadherin expression. Data are presented as the mean ± SEM; two-tailed unpaired Student’s t test; *P < 0.05 and **P < 0.01

Members of the matrix metalloproteinase (MMP) family promote cell migration. Moreover, MMP gene expression plays a key role in cancer metastasis, neurological injury and disease, and development [22–26]. Among these, MMP2 and MMP9 are gelatinases [27] that contribute to glioma progression [28, 29]. Therefore, we investigated the effects of Calanquinone A on MMP2 and MMP9 expression in glioma cells. We revealed that Calanquinone A treatment downregulated MMP9 expression in Pt#3 and U87MG cells but had no effect on MMP2 expression (Fig. 4A–D). Furthermore, the epithelial-mesenchymal transition (EMT) phenotype in cancer cells promotes tumor metastasis and is triggered by the downregulation of E-cadherin or upregulation of N-cadherin [30–32]. Thus, we investigated whether Calanquinone A affects cadherin protein expression and found that Calanquinone A treatment upregulated E-cadherin expression in Pt#3 and U87MG cells; however, N-cadherin expression did not significantly change (Fig. 4E–G). These data suggest that Calanquinone A inhibits cell migration by downregulating MMP9 and upregulating E-cadherin expression.

### Calanquinone A effectively inhibited the STAT3 transcription factor in glioma cells

**Fig. 5:**
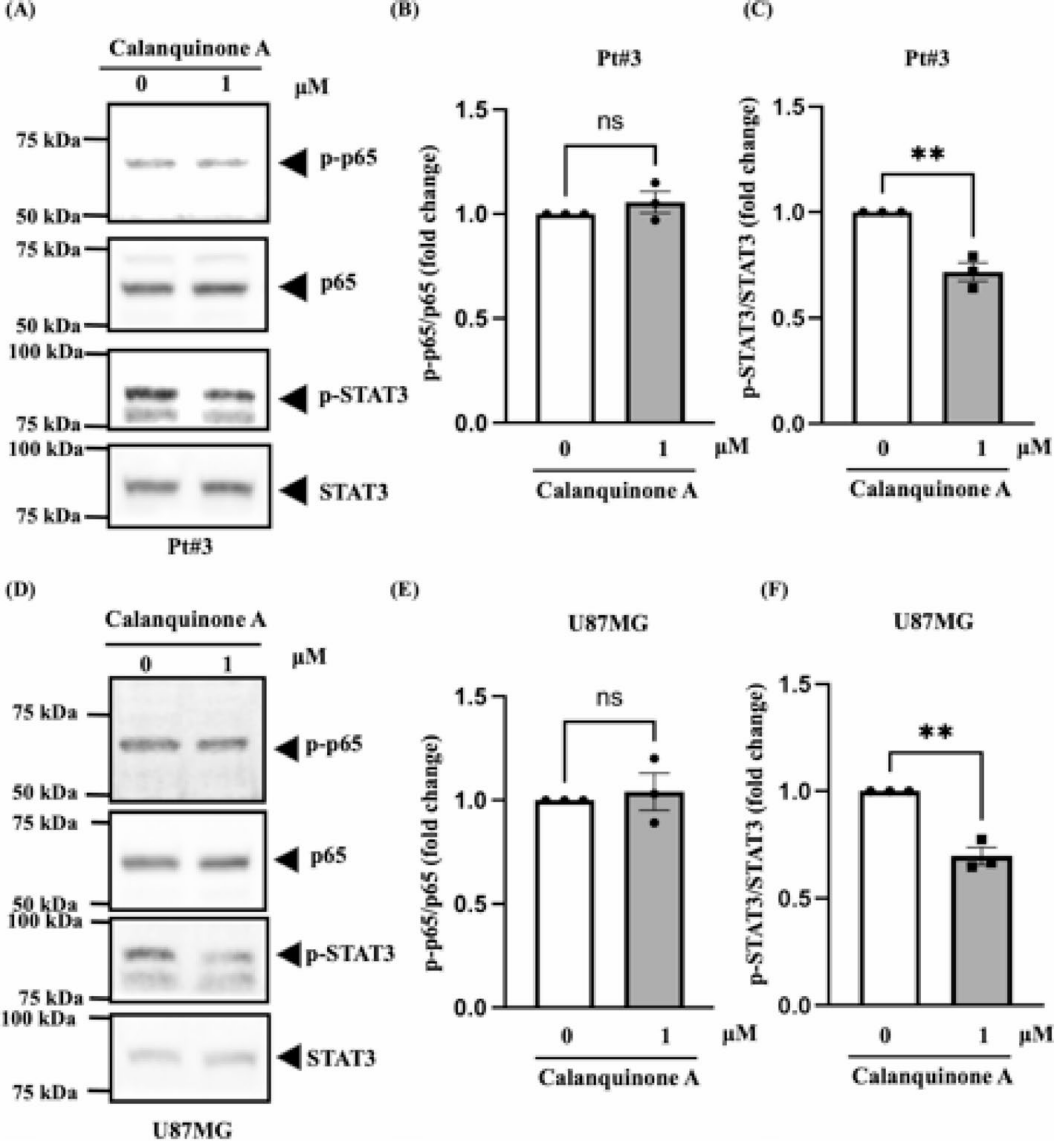
Calanquinone A inactivated the STAT3 transcription factor in glioma cells. **A** Calanquinone A reduced STAT3 phosphorylation in Pt#3 cells without affecting p65 activation. **B** and **C** Statistical analysis of the results from **A** confirmed that Calanquinone A had no effect on p65 phosphorylation but significantly reduced phospho-STAT3 (Tyr705) activation. **D** Similarly, treatment of U87MG cells with Calanquinone A reduced STAT3 phosphorylation without affecting p65 activation. **E** and **F** Statistical analysis of the results from **D** confirmed that Calanquinone A significantly reduced STAT3 activation but did not affect p65 activation. Data are presented as the mean ± SEM; two-tailed unpaired Student’s t test; **P < 0.01

STAT3 and NF-ĸB have been found to directly regulate the expression of c-Myc and MMP9 in glioma cells [33–36]. However, it remains unclear whether Calanquinone A inhibits STAT3 or p65 activation in glioma cells. Calanquinone A treatment reduced phospho-STAT3 (Tyr705) activation but did not affect phospho-p65 activation in Pt#3 and U87MG cells (Fig. 5). Furthermore, we also examined MAPK pathway activation following Calanquinone A treatment. The data showed that Calanquinone A did not affect MAPK pathway activity (Supplementary Fig. 1). These data revealed that Calanquinone A may inhibit c-Myc and MMP9 expression by suppressing STAT3 pathway activity.

### STAT3-induced c-Myc and MMP9 expression, thereby reducing glioma cell proliferation and migration

**Fig. 6:**
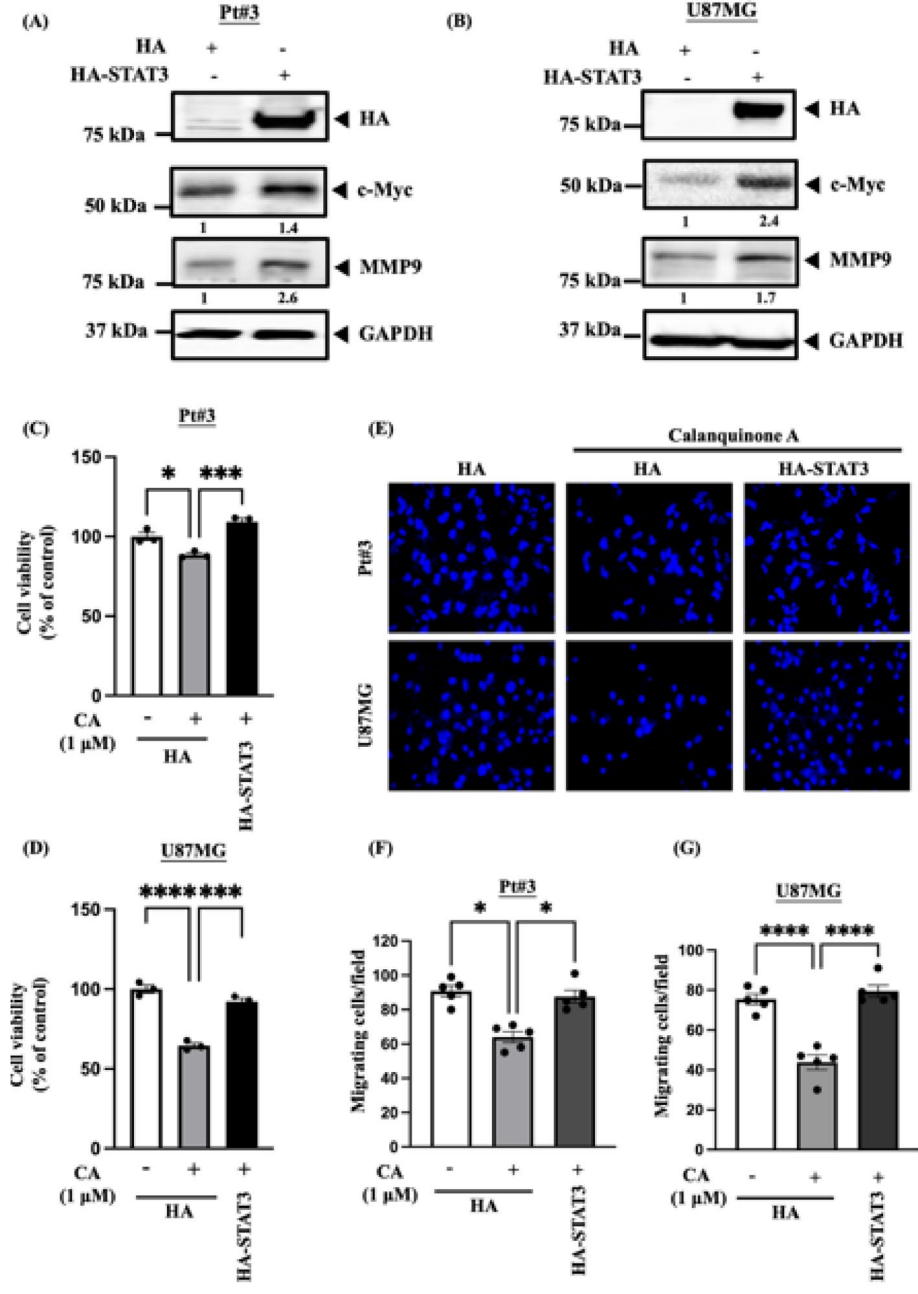
Calanquinone A reduced glioma proliferation and migration through the STAT3/c-Myc and STAT3/MMP9 pathways. Overexpression of STAT3 in **A** Pt#3 and **B** U87MG cells increased c-Myc and MMP9 protein expression. Some blots were cut prior to antibody hybridization to allow probing of different proteins on the same membrane; therefore, full-length blot images are not available for certain targets. Additionally, due to low exposure levels, membrane edges are not visible for GAPDH. The available cut, uncropped blots are provided in the Supplementary Information. **C** and **D** STAT3 overexpression mitigated Calanquinone A-induced cell death in Pt#3 and U87MG cells. **E** STAT3 overexpression reversed Calanquinone A-induced suppression of cell migration in Pt#3 and U87MG cells. **F** and **G** Quantification of migration in Pt#3 and U87MG cells showed that STAT3 overexpression counteracts Calanquinone A-induced reduction in cell migration. Data were analyzed using one-way ANOVA with Prism software. *P < 0.05, ***P < 0.001, and ****P < 0.0001

First, we examined whether STAT3 overexpression regulates c-Myc and MMP9 expression in Pt#3 and U87MG cells, and our results confirmed that STAT3 overexpression indeed elevated c-Myc and MMP9 expression in these cells (Fig. 6A, B). We further investigated whether STAT3 expression reversed the effects of Calanquinone A on glioma cell proliferation and migration. Our results showed that STAT3 overexpression compensated for Calanquinone A-induced cell death (Fig. 6C, D). Additionally, STAT3 expression increased cell migration compared to that in the control groups treated with Calanquinone A (Fig. 6E–G). Calanquinone A inhibits glioma cell proliferation and migration by targeting the STAT3/c-Myc and STAT3/MMP9 signaling pathways.

### Calanquinone A directly binds to STAT3 and suppresses tumor growth in vivo

**Fig. 7:**
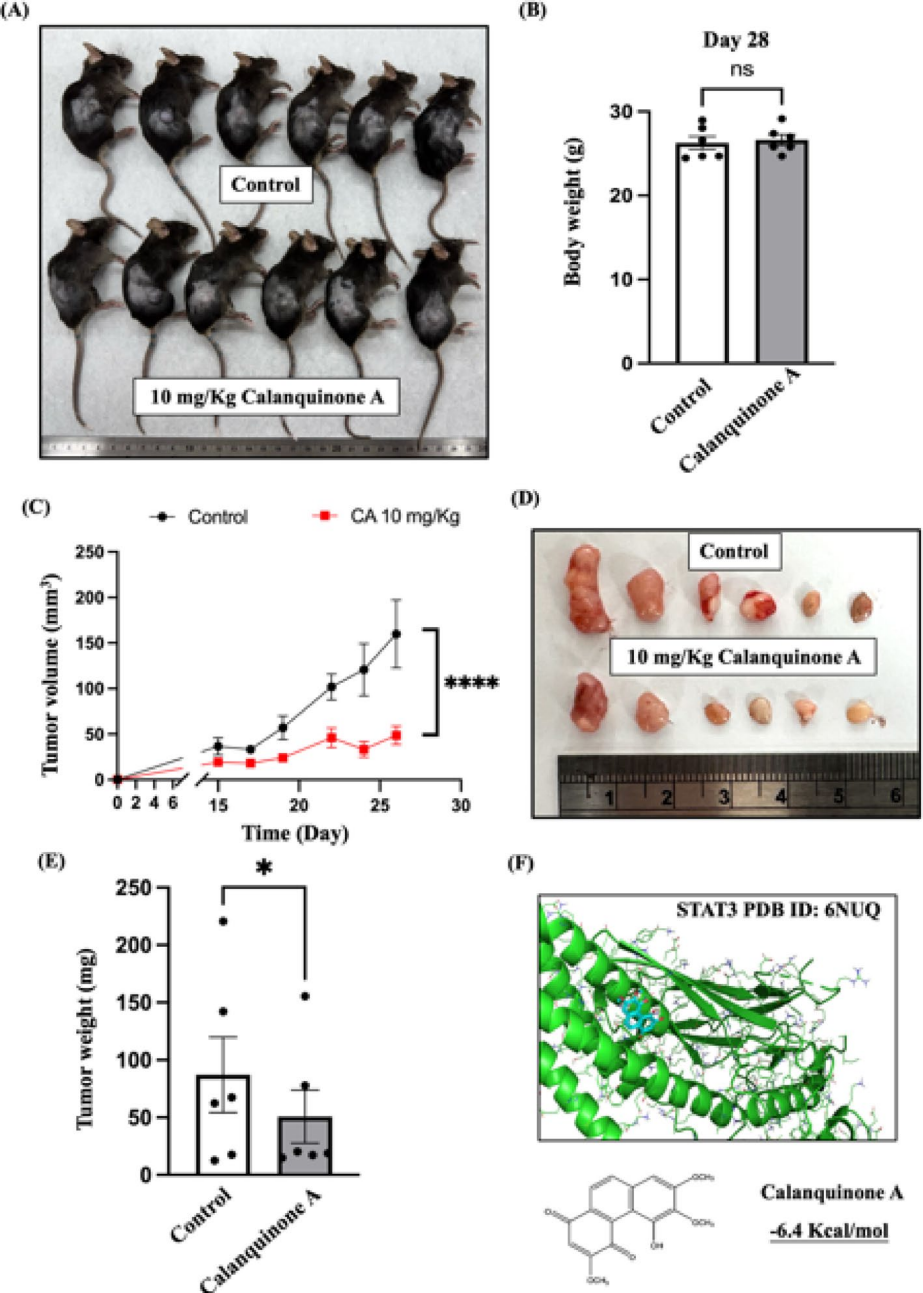
Calanquinone A reduces glioma tumorigenesis by directly binding to STAT3. **A** Schematic diagram illustrating Calanquinone A-mediated suppression of tumor formation in a murine model. **B** Calanquinone A treatment did not significantly affect body weight in G261-inoculated tumor-bearing mice. Data are presented as the mean ± SEM; two-tailed unpaired Student’s t test; ns: not significant. **C** Tumor volume was significantly reduced following Calanquinone A treatment, as determined by non-linear regression with best-fit analysis (****P < 0.0001). **D** Representative images of excised tumors and **E** quantification of tumor weights show reduced tumor burden in the treatment group. Data are presented as the mean ± SEM; two-tailed paired Student’s t test; *P < 0.05. **F** Molecular docking analysis revealed four potential binding pockets between Calanquinone A and STAT3 (PDB ID: 6NUQ), with a binding energy of − 6.4 kcal/mol, indicating a favorable interaction

**Fig. 8:**
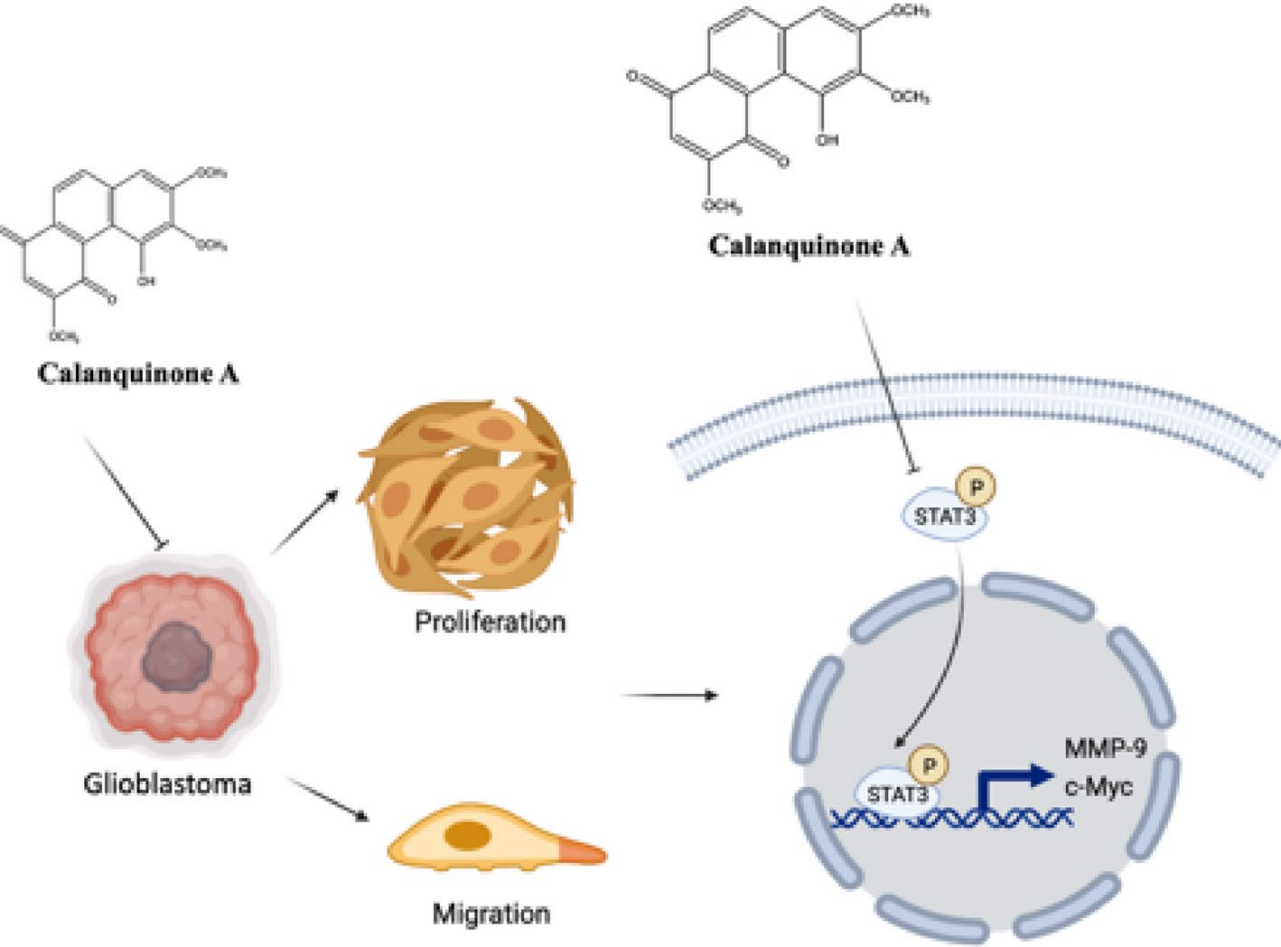
Proposed model of Calanquinone A-mediated inhibition of glioma progression via STAT3/c-Myc and STAT3/MMP9 pathways. Calanquinone A reduces both short- and long-term proliferation and cell migration. The underlying molecular mechanisms involve the STAT3/c-Myc and STAT3/MMP9 pathways. This proposed mechanism illustrates the potential of Calanquinone A as a multi-targeted natural agent for cancer prevention or adjunct therapy through dietary means

Based on the aforementioned findings, Calanquinone A reduces glioma tumorigenesis in cell-based models. However, its effect on tumor formation in vivo had not been established. To investigate this, G261 glioma cells were subcutaneously inoculated into the right hind flank of mice. Our results showed that Calanquinone A did not affect body weight (Fig. 7A, B) but significantly reduced both tumor volume (Fig. 7C) and tumor weight (Fig. 7D, E). Furthermore, molecular docking analysis revealed four potential binding sites between Calanquinone A and STAT3, with a binding energy of − 6.4 kcal/mol (Fig. 7F). Collectively, these results demonstrate that Calanquinone A suppresses glioma proliferation and migration by targeting the STAT3/c-Myc and MMP9 pathways (Fig. 8).

## Discussion

Calanquinone A has been demonstrated to exhibit anti-glioma effects; however, the detailed molecular mechanism underlying this remains unclear [13]. In this present study, we demonstrated that Calanquinone A suppressed the survival, proliferation, and migration of GBM cells. Additionally, we elucidated the underlying molecular mechanisms by revealing that Calanquinone A treatment decreases MMP9 and c-Myc expression in GBM cells. Moreover, this effect was not mediated by p65, but rather through STAT3 (Fig. 8).

Species of the genus Calanthe have been traditionally used in Asian medicine and are known to contain diverse bioactive phytochemicals, including alkaloids, terpenoids, and phenolic compounds. These constituents have been associated with a range of pharmacological activities, such as anticancer, anti-inflammatory, antiarthritic, antidiabetic, and antibacterial effects [14]. Notably, Calanquinone A, a major bioactive compound isolated from Calanthe species, has demonstrated antitumor activity [13, 37]. Calanquinone A has a molecular weight of 314.29 g/mol, which is within the range typically associated with compounds capable of penetrating the blood–brain barrier (i.e., < 400 Da) [15]. In addition to its therapeutic potential, Calanquinone A may be considered a promising candidate for development as a therapeutic agent for glioma. Calanthe arisanensis, the botanical source of Calanquinone A, has a well-documented history of ethnobotanical use in East Asia [14], particularly in traditional decoctions and medicinal teas, suggesting a culturally relevant route for oral consumption. Although Calanquinone A has not yet been fully characterized in terms of its dietary abundance or food stability, its moderate molecular weight and lipophilic structure are indicative of favorable gastrointestinal absorption and bioavailability. Future research should focus on evaluating its pharmacokinetics, formulation stability, and extraction efficiency in food-compatible systems to enable its potential integration into dietary or nutraceutical applications. Notably, C. arisanensis has been shown to yield Calanquinone A in quantifiable amounts, supporting the feasibility of its scalable extraction and standardization for potential preventive applications [37]. These considerations are consistent with the increasing interest in food-derived phytochemicals for promoting brain health and preventing cancer.

A previous study indicated that c-Myc is deregulated in most GBM cells and is associated with poor prognosis in patients with tumors [38]. Both in vitro and in vivo studies have confirmed that Myc inhibition suppresses glioma formation and inhibits glioma cell proliferation and survival [39]. Mechanistically, c-Myc interacts with Myc-associated factor X (MAX), and the complex binds to promoters of downstream genes associated with cell survival and proliferation, such as Bcl-2 and CDK4, and modulates their expression [40]. Taken together, these results indicated that c-Myc plays a key role in GBM progression and confirmed that Calanquinone A effectively suppresses c-Myc expression and reduces the viability of GBM cells (Figs. 1 and 3). In addition, c-Myc inhibition has been demonstrated to sensitize resistant recurrent GBM2 cells to treatment [41]; therefore, Calanquinone A may also combat TMZ chemoresistance in GBM.

MMPs are enzymes secreted by cells or bound to the plasma membrane. They participate in several physiological processes such as angiogenesis, tissue repair, cell invasion, and cell migration [42]. Specifically, both MMP2 and MMP9 are gelatinases that can break down type IV collagen in the basement membrane, thereby promoting the metastasis of GBM cells [42, 43]. Elevated expression of MMP2/9 indicates poor prognosis in glioma recurrence [28]. In addition to MMPs, EMT also contributes to tumor metastasis [30, 44]. E-cadherin plays a critical role in maintaining epithelial cell adhesion and preventing the initial detachment of cells from the primary tumor mass. When cell-cell adhesion and junctions are disrupted, epithelial cells lose their structural integrity, enabling them to invade surrounding tissues and migrate to distant locations [45]. As shown in Fig. 2, Calanquinone A notably decreased the number of migrating GBM cells, significantly upregulated E-cadherin expression, and reduced MMP9 expression (Fig. 4). These results indicate that Calanquinone A suppresses the migration of GBM cells through E-cadherin-mediated cell-cell adherence and MMP9 expression.

We verified that Calanquinone A downregulated STAT3 protein expression and that inhibiting STAT3 reduces GBM cell proliferation and metastasis (Fig. 5) [4, 46]. STAT3 has been shown to modulate the expression of c-Myc and MMP9 [33–36]. Overexpression of STAT3 reversed the decrease in the migratory ability and viability of GBM cells induced by Calanquinone A treatment (Fig. 6). IL-6 is an inflammatory mediator that also activates STAT3 and promotes tumor progression [47]. Notably, patients with GBM with IL-6 gene amplification exhibit significantly shorter survival than those without this amplification [47]. Therefore, these results suggest that Calanquinone A may inhibit GBM development by counteracting the effects of IL-6 during inflammation [47]. Although the inhibition of STAT3 phosphorylation and the downregulation of c-Myc and MMP9 are well-documented mechanisms targeted by numerous phytochemicals, our study highlights several aspects that contribute to its novelty and scientific significance: (1) Calanquinone A directly binds to STAT3, as supported by molecular docking analysis; (2) we utilized patient-derived primary GBM cells (Pt#3) to assess the functional effects of Calanquinone A; and (3) we employed an in vivo xenograft model to evaluate the therapeutic efficacy of Calanquinone A in glioblastoma.

STAT3 has been reported to regulate a range of stemness-associated transcription factors, including Nanog, Sox2, and Oct4, in glioblastoma, thereby facilitating the formation and maintenance of glioma stem-like cells (GSCs) [48–50]. These GSCs contribute to tumor initiation, recurrence, and resistance to therapy. In our study, we found that Calanquinone A markedly reduces STAT3 phosphorylation, a modification critical for its activation and nuclear translocation. This raises the possibility that Calanquinone A may also interfere with the stemness properties of glioma cells through STAT3 inhibition. Future investigations are warranted to examine whether Calanquinone A can reduce the expression of stemness markers and impair the self-renewal capacity of GSCs using neurosphere assays and limiting dilution transplantation models.

Moreover, STAT3 exerts its transcriptional functions primarily through nuclear translocation. Nucleocytoplasmic transport, including both passive diffusion and active transport mechanisms via importins, is essential for the nuclear import of STAT3 and other transcription factors [51]. Disruption of this process has been shown to impair STAT3’s transcriptional activity and downstream oncogenic signaling. While our molecular docking results support a direct interaction between Calanquinone A and STAT3, it remains unclear whether this compound also interferes with STAT3 nuclear localization. Investigating whether Calanquinone A blocks STAT3 translocation into the nucleus warrants further exploration in future studies.

In conclusion, our study demonstrates that Calanquinone A exerts potent antitumor effects against glioblastoma multiforme by inhibiting cell viability, proliferation, and migration through suppression of the STAT3/c-Myc and STAT3/MMP9 signaling pathways. In vivo findings and molecular docking analyses further support a direct interaction between Calanquinone A and STAT3, suggesting a specific molecular mechanism of action. Notably, Calanquinone A is a natural phytochemical derived from Calanthe arisanensis, a traditional medicinal orchid with a long history of dietary and ethnopharmacological use in East Asia. With its favorable molecular weight for blood–brain barrier permeability and lipophilic chemical properties, Calanquinone A shows potential as a therapeutic agent for promoting brain health and preventing glioma. Together, these findings underscore the potential of integrating traditional medicinal compounds into modern translational strategies for cancer therapy and health promotion.

This study demonstrates that Calanquinone A effectively inhibits glioma cell proliferation and migration by targeting the STAT3/c-Myc and MMP9 signaling pathways. However, several important limitations should be acknowledged. First, although molecular docking suggests a potential interaction, our current data do not provide direct biochemical evidence confirming Calanquinone A binding to the STAT3 protein. Second, while we emphasize the inhibitory effects of Calanquinone A on glioma cells, its impact on non-glioma (normal) cells was not investigated, due to limited access to human primary non-tumor glial cell lines and technical constraints. Third, we recognize that the redox-active nature of quinone compounds like Calanquinone A may modulate additional molecular pathways beyond STAT3. These alternative mechanisms, such as redox-sensitive signaling pathways, could contribute to the observed anti-glioma effects and warrant further investigation in future studies.

## Supplementary Information

Below is the link to the electronic supplementary material.

Supplementary Material 1

Supplementary Material 2

## Data Availability

The data that support this study are available from the corresponding authors upon reasonable request.

## Abbreviations

CA: Calanquinone A
MMP: Matrix metalloproteinase
DAPI: 4′,6-Diamidino-2-phenylindole
PVDF: Polyvinylidene difluoride
SDS-PAGE: Sodium dodecyl sulfate-polyacrylamide gel electrophoresis
OD: Optical density
GSCs: glioma stem-like cells
