# Supplementary material for: Calanquinone A suppresses glioma progression via STAT3-mediated regulation of c-Myc and MMP9
Source: Discov Oncol. 2025 Aug 4;16:1463. doi: 10.1007/s12672-025-03279-4 (PMC12321724; doi:10.1007/s12672-025-03279-4)

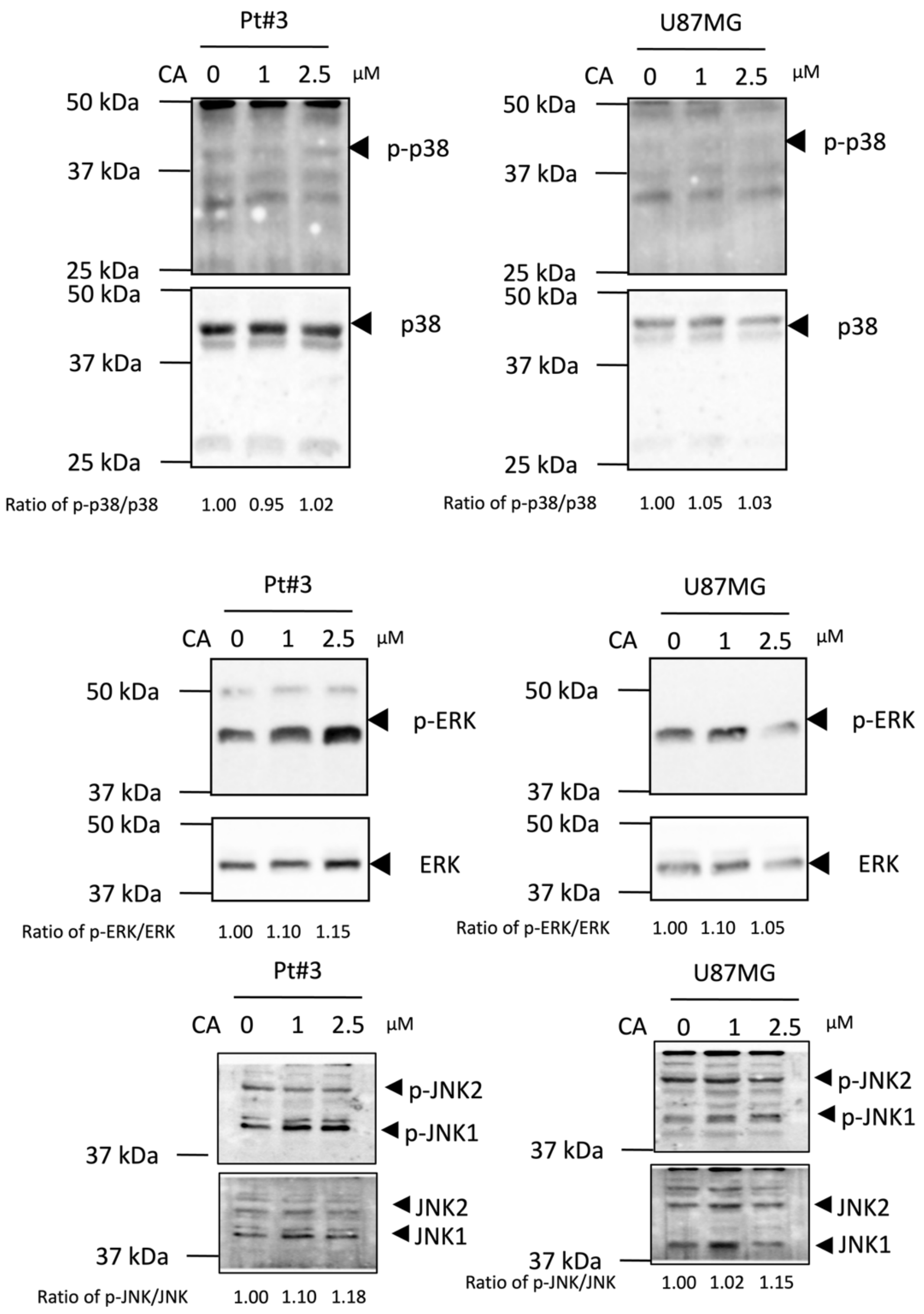

Supplementary Figure 1. Calanquinone A does not alter MAPK pathway activity in glioma cells. To determine whether Calanquinone A influences the MAPK signaling pathway, we examined the phosphorylation status of ERK1/2, JNK, and p38 in glioma cells treated with increasing concentrations of Calanquinone A. Western blot analysis revealed that the levels of phospho-ERK1/2, phospho-JNK, and phospho-p38 remained unchanged upon Calanquinone A treatment, indicating that this compound does not significantly impact MAPK pathway activation. Total protein levels of ERK1/2, JNK, and p38 were also unaffected.

Figure 3 (A) and (B) show the whole blot after cutting membrane at the molecular weight 50 kDa and 37 kDa for c-Myc (52 kDa) and GAPDH (37 kDa)

(A)

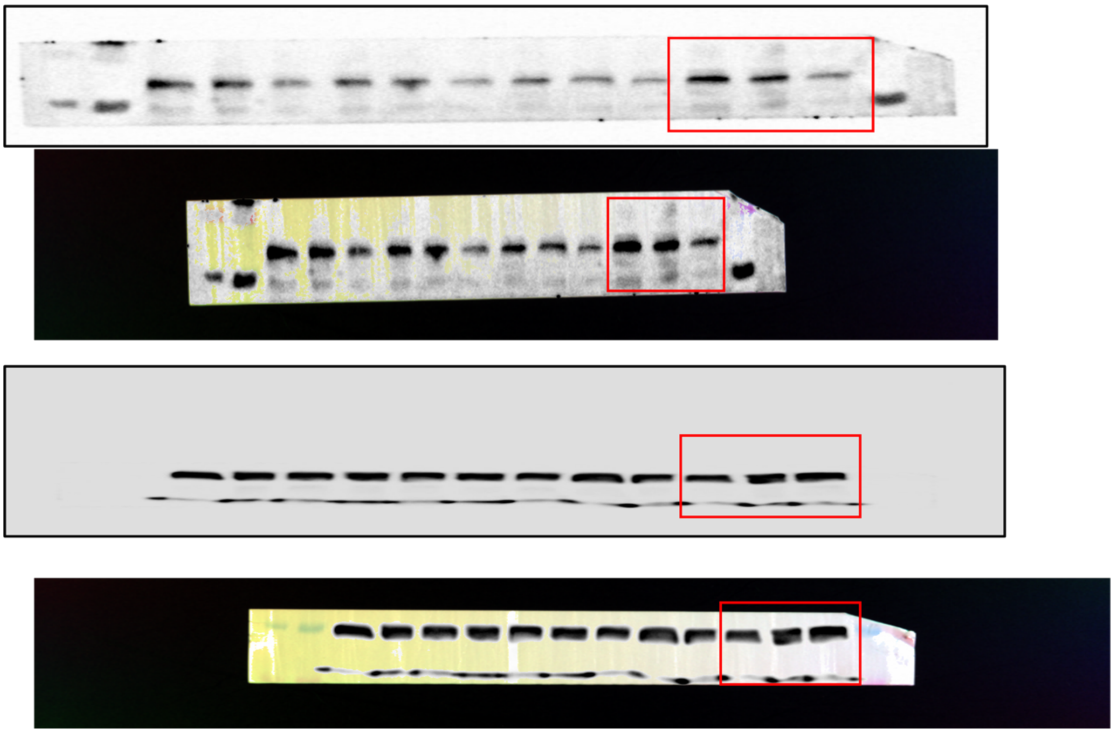

(B)

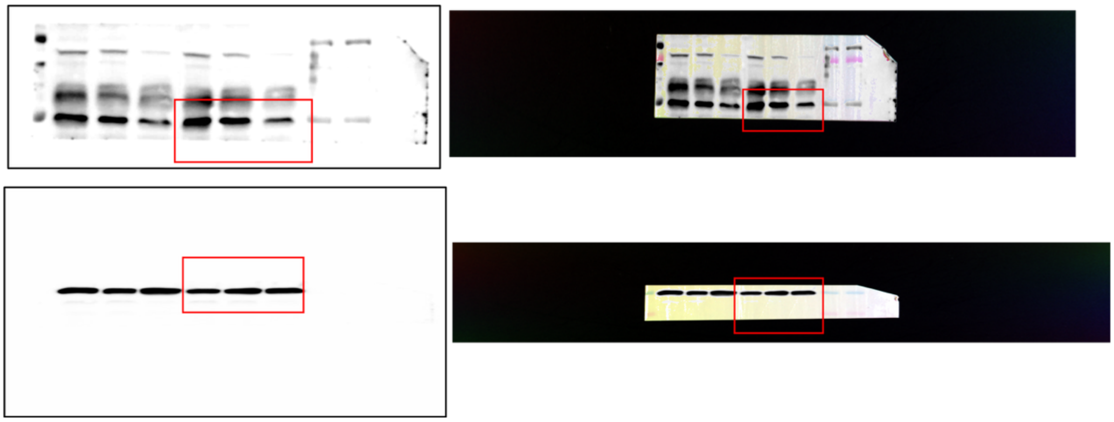

Figure 4 (A), (C), (E), and (F) show the whole blot after cutting membrane at molecular weights 150 kDa 100 kDa, 75 kDa, 50 kDa, and 37 kDa for MMP2 (66kDa), MMP9 (92 kDa), E-cadherin (120 kDa), N-cadherin (150 kDa), and GAPDH (37 kDa)

(A)

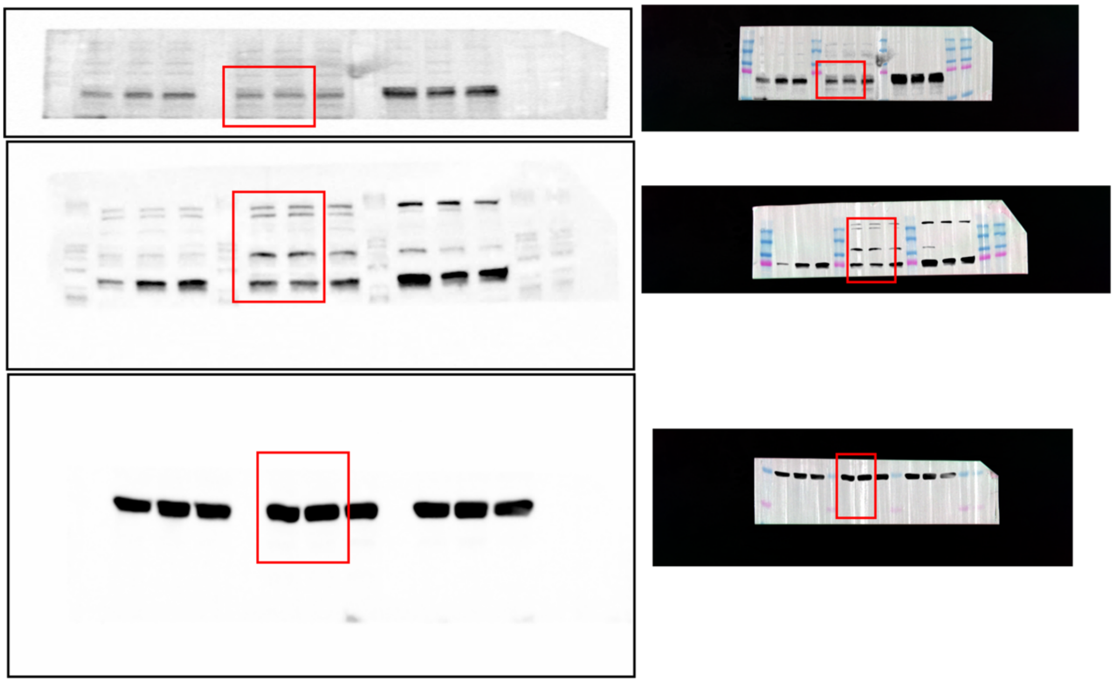

(C)

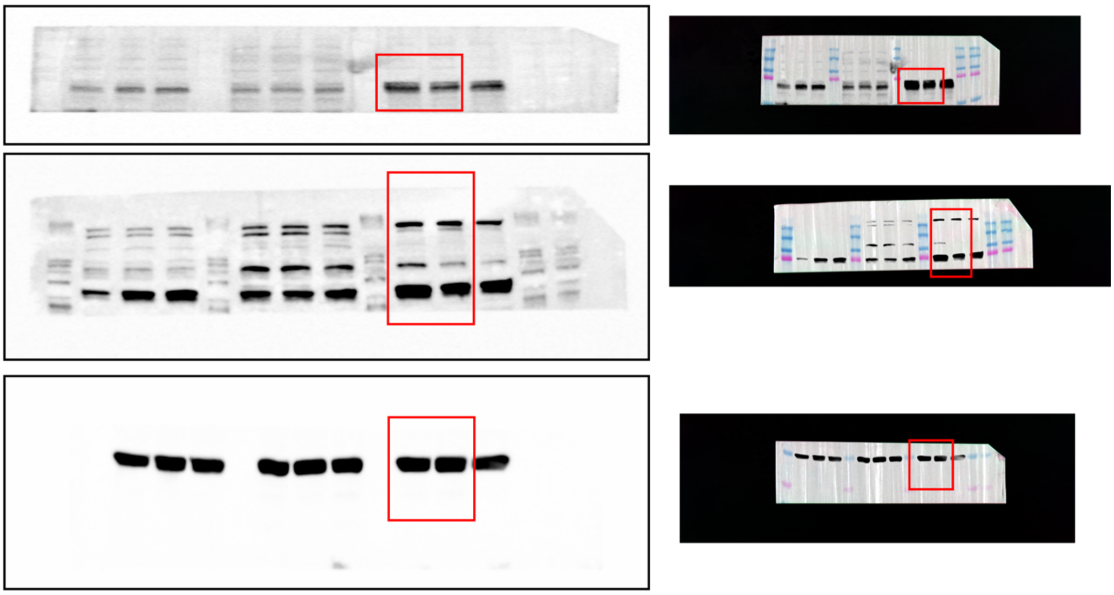

(E)

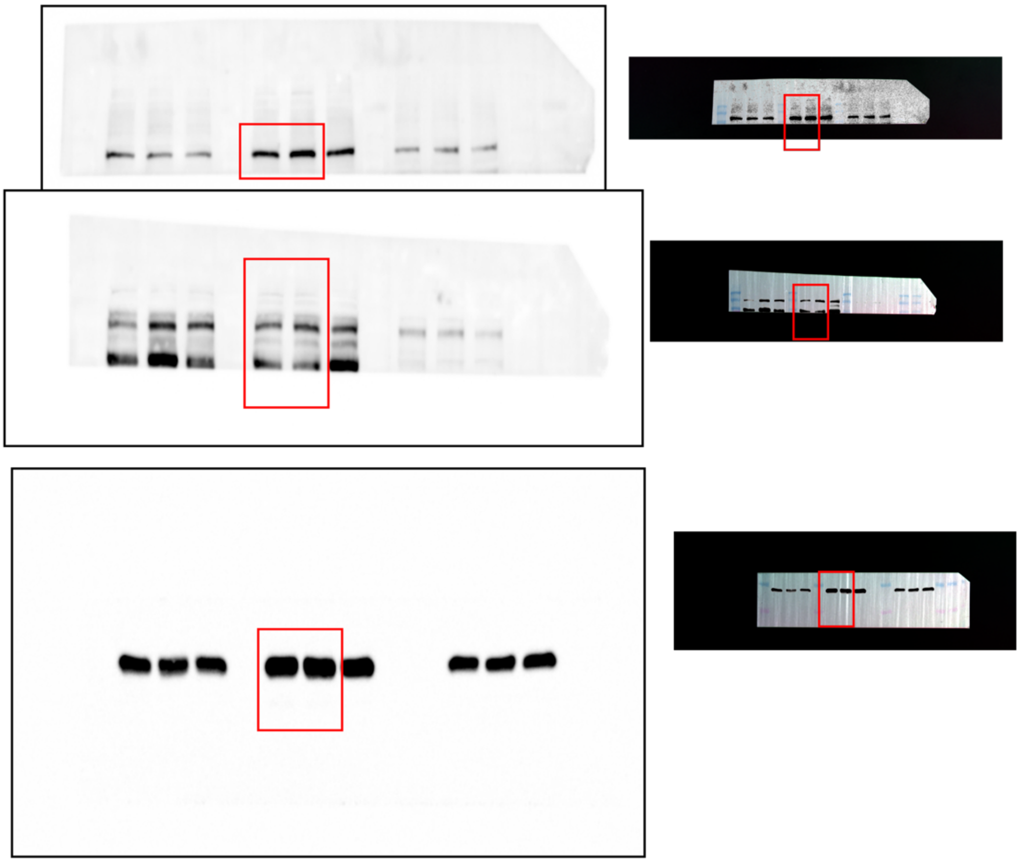

(F)

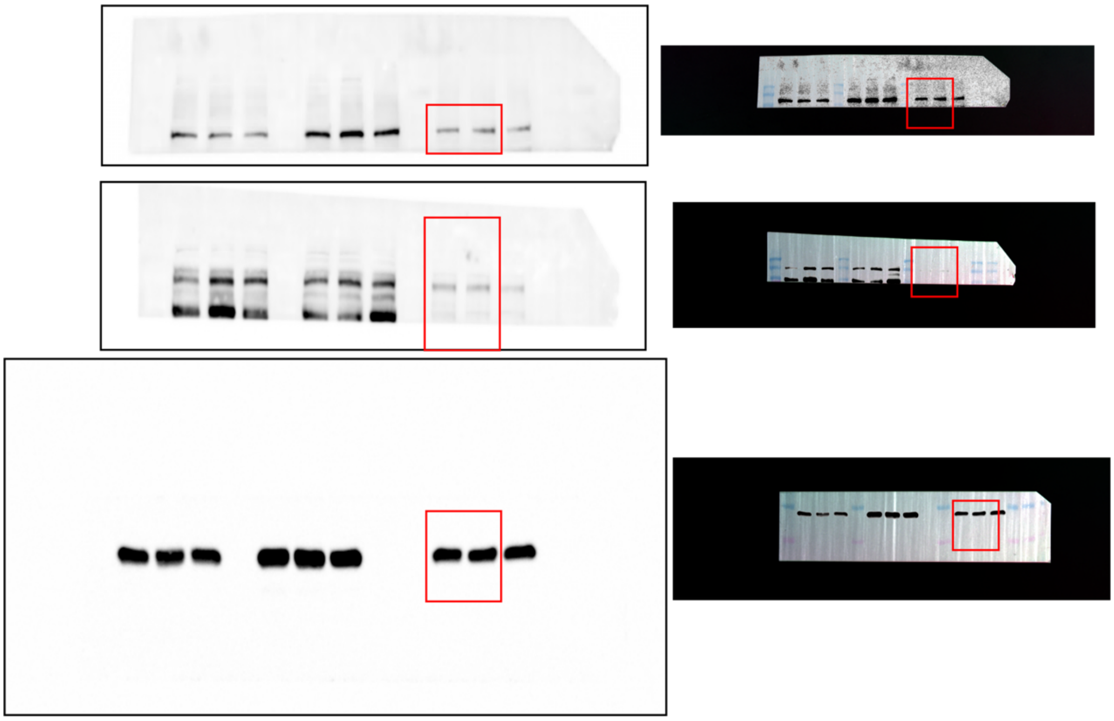

Figure 5 (A) and (D) show the whole blot after cutting the membrane at the molecular weights 100 kDa, 75 kDa, and 50 kDa for p-p65 (65 kDa), p65 (65 kDa), p-STAT3 (87 kDa), and STAT3 (87 kDa)

(A)

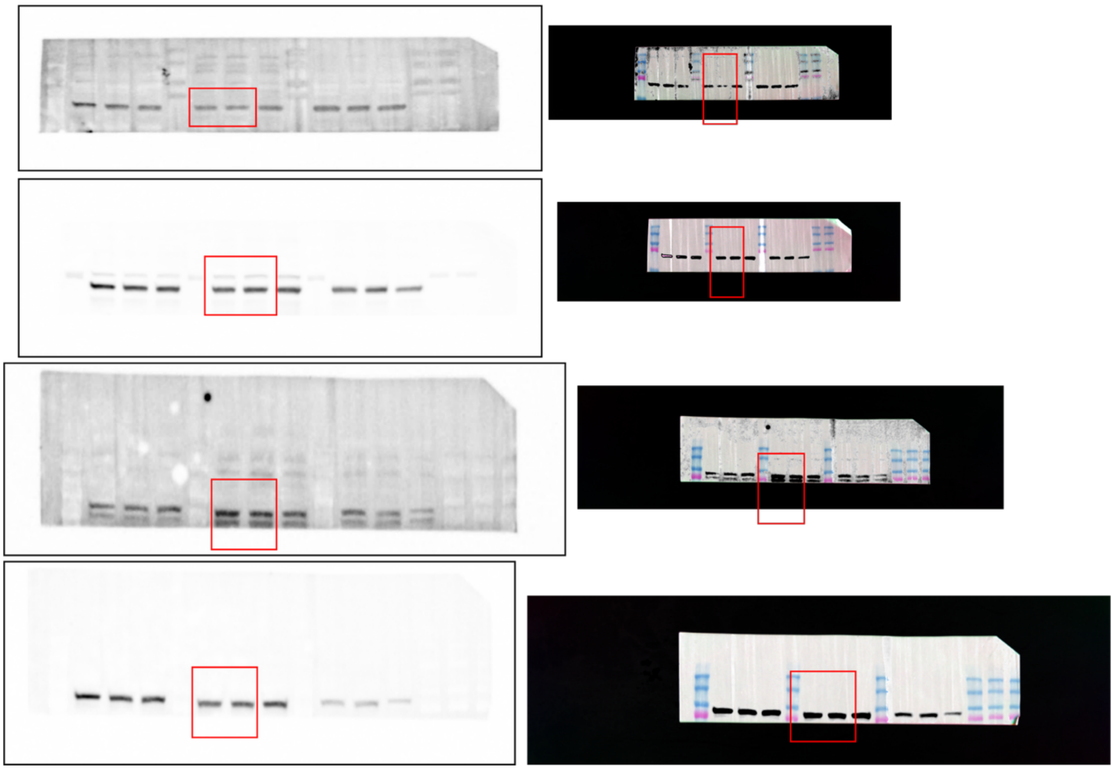

(D)

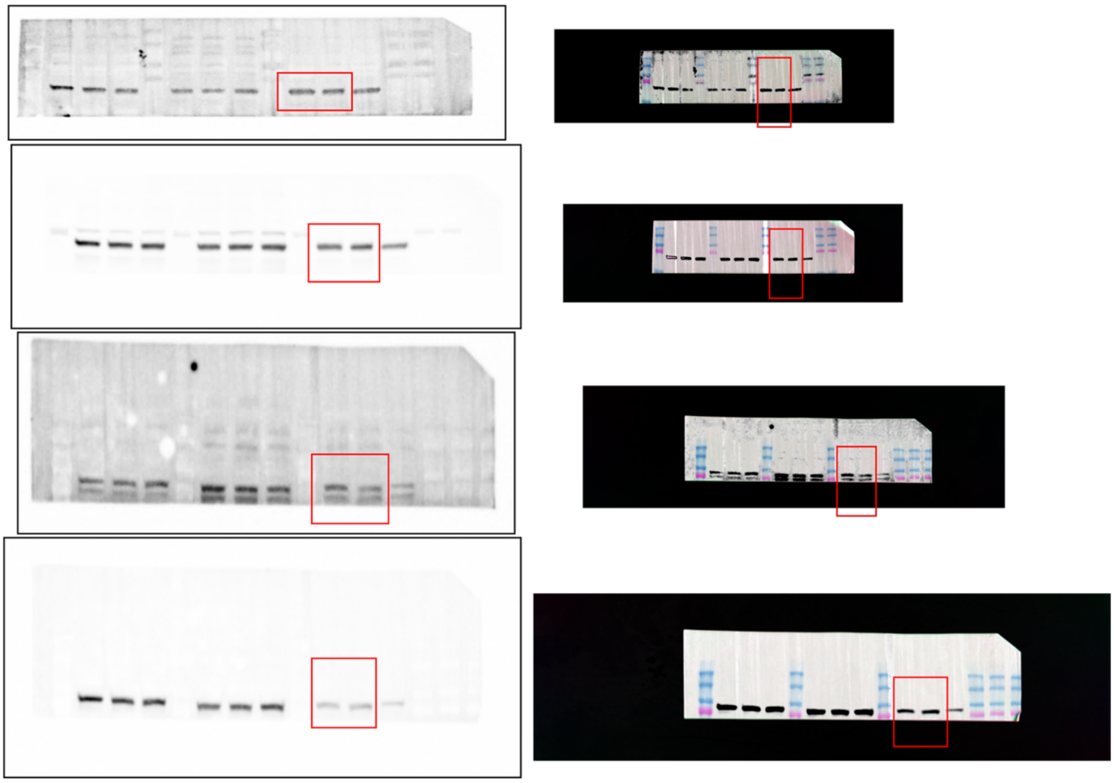

Figure 6 (A) and (B) show the whole blot after cutting the membrane at the molecular weights 75 kDa, 50 kDa, and 37 kDa for HA-STAT3 (87 kDa), c-Myc (52 kDa), MMP9 (92 kDa), and GAPDH (37 kDa)

(Note: Some blots were cut prior to antibody hybridization to allow probing of different proteins on the same membrane; therefore, full-length blot images are not available for certain targets. Additionally, due to low exposure levels, membrane edges are not visible for GAPDH. The available cut, uncropped blots are provided in the Supplementary Information.)

(A)

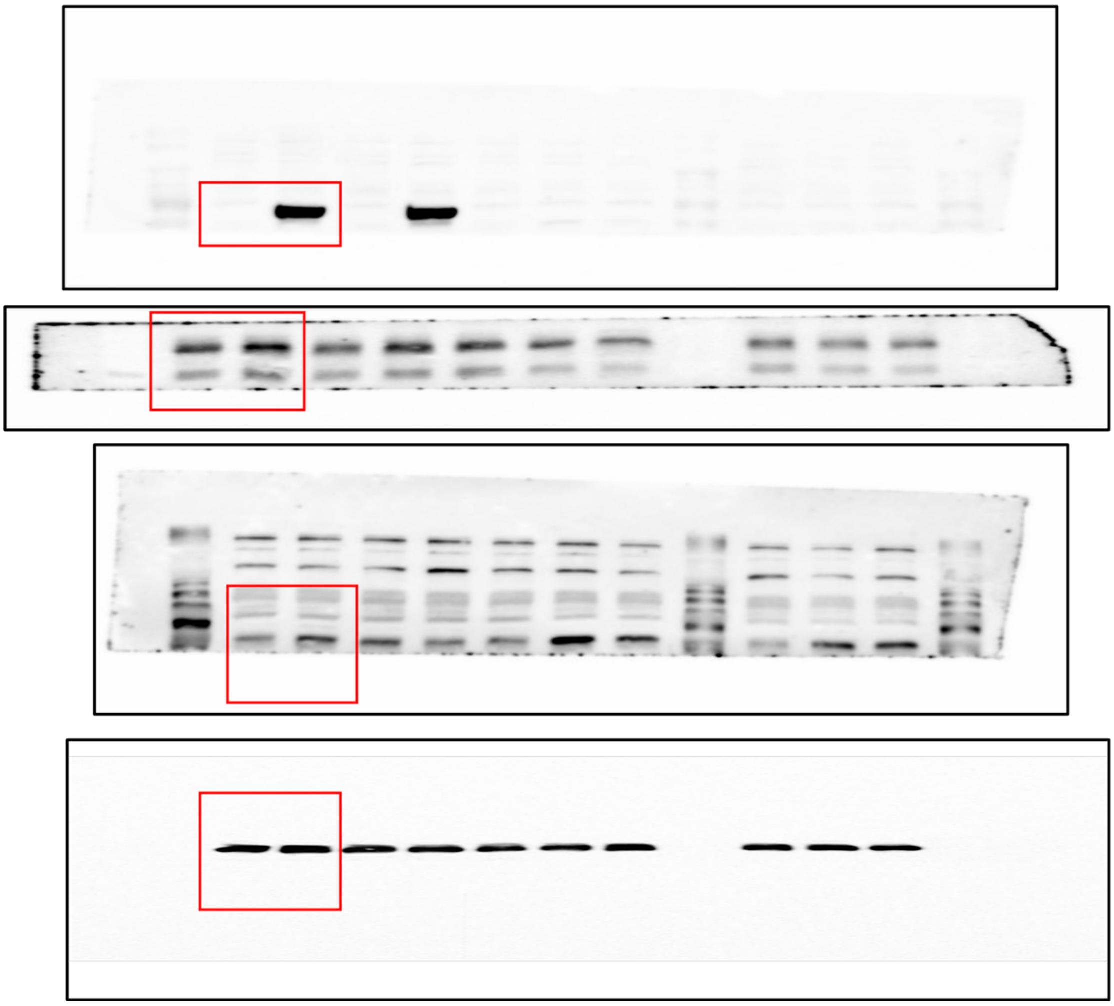

(B)

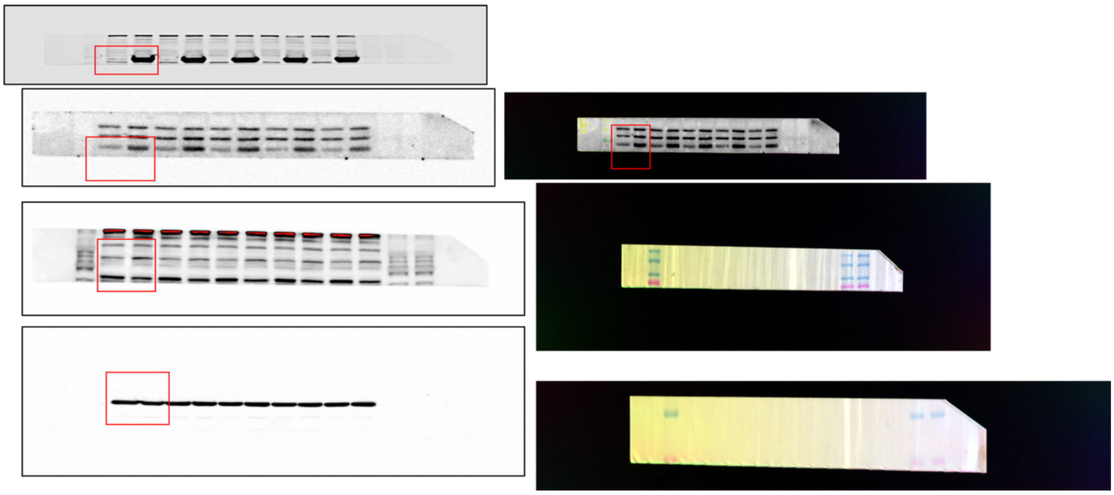

Supplementary Figure 1 show the whole blot after cutting the membrane at the molecular weights 50 kDa~25 kDa for p-p38, p38, p-ERK, ERK, p-JNK1/2, and JNK1/2

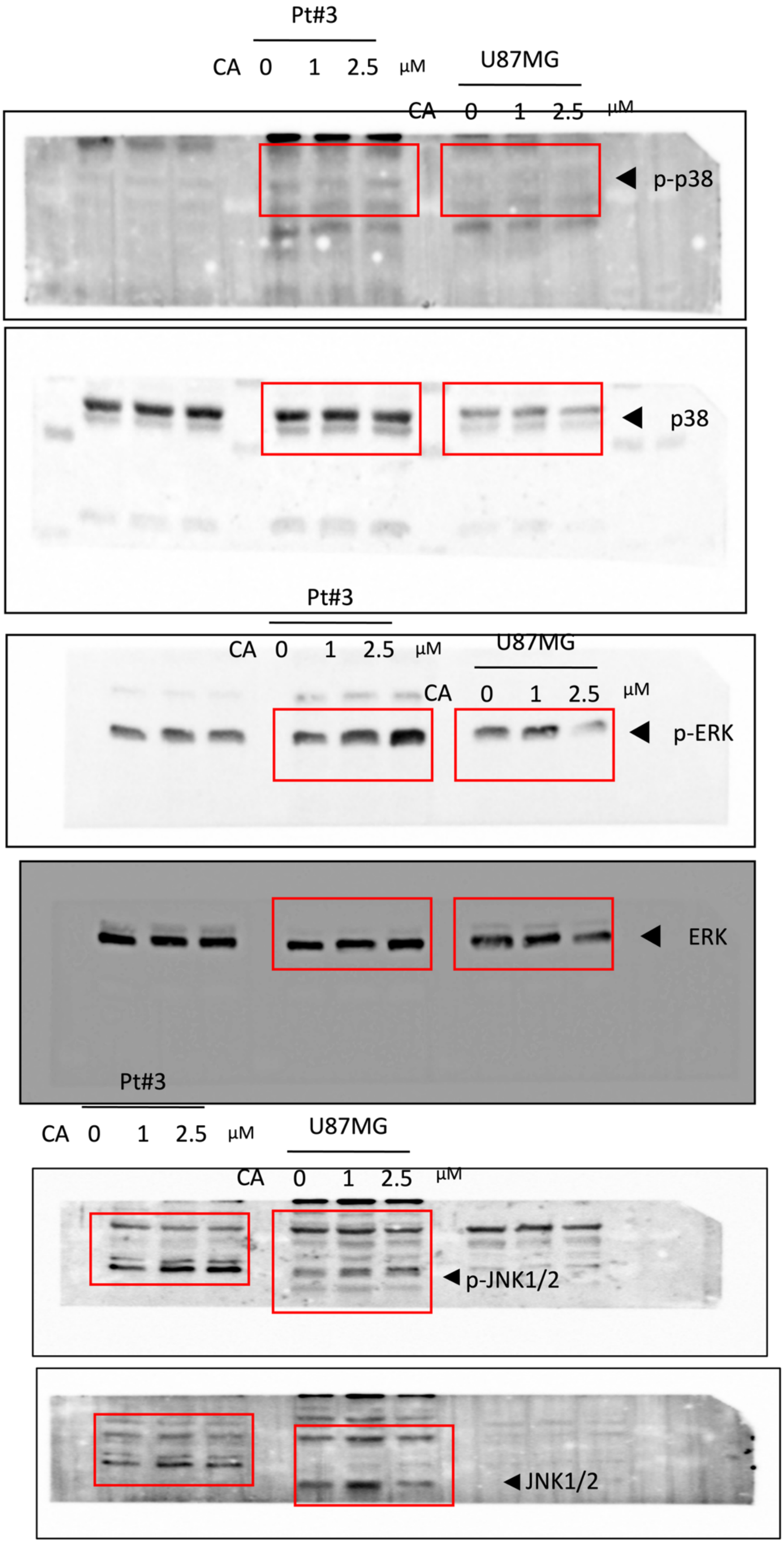

Supplement: Supplementary file 1 — Supplementary Material 1 [file 12672_2025_3279_MOESM1_ESM.pdf]
