# Supplementary material for: Calanquinone A suppresses glioma progression via STAT3-mediated regulation of c-Myc and MMP9
Source: Discov Oncol. 2025 Aug 4;16:1463. doi: 10.1007/s12672-025-03279-4 (PMC12321724; doi:10.1007/s12672-025-03279-4)
Supplement: Supplementary file 2 — Supplementary Material 2 [file 12672_2025_3279_MOESM2_ESM.docx]

**
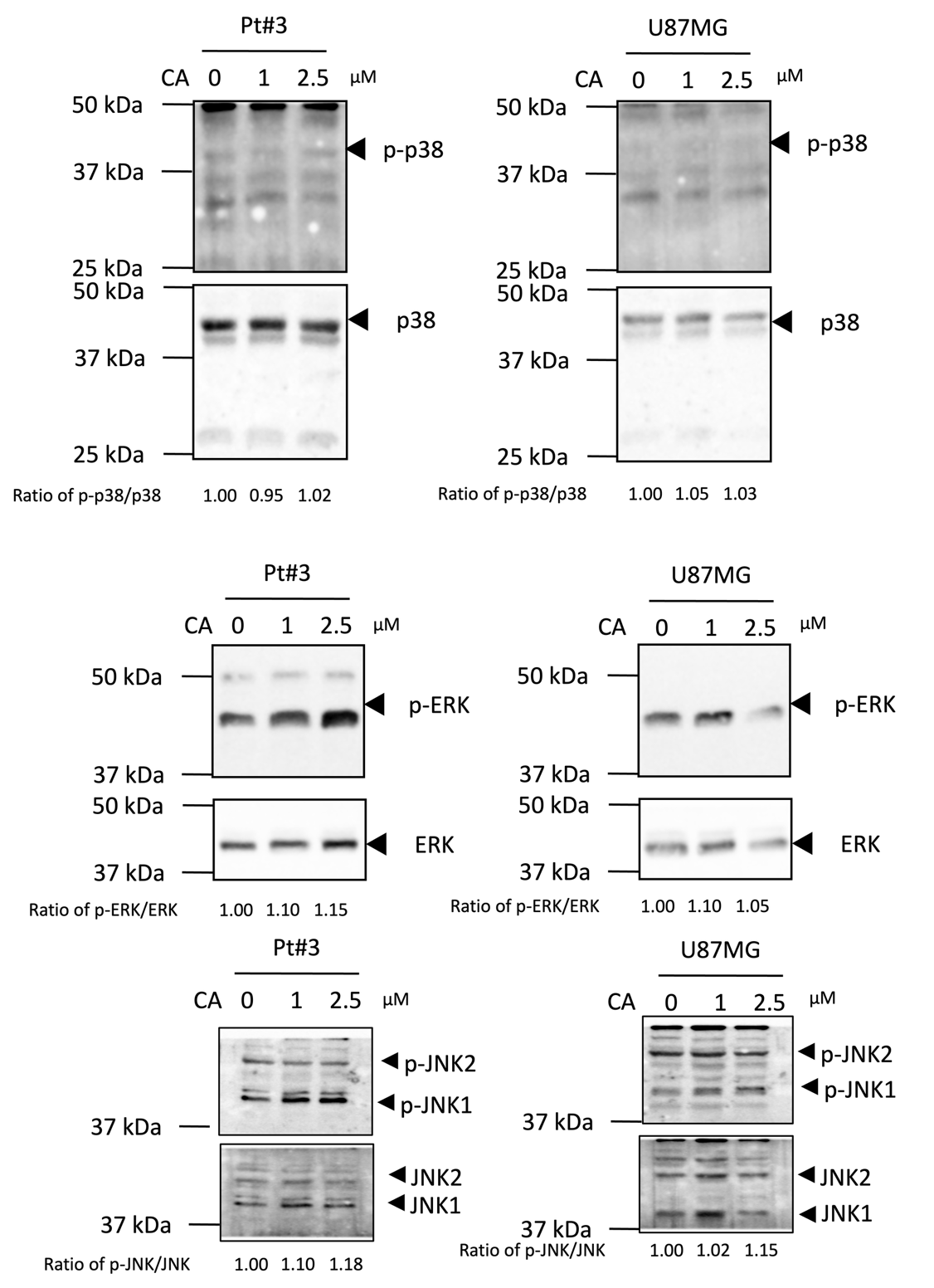
**

**Supplementary Figure 1.** Calanquinone A does not alter MAPK pathway activity in glioma cells. To determine whether Calanquinone A influences the MAPK signaling pathway, we examined the phosphorylation status of ERK1/2, JNK, and p38 in glioma cells treated with increasing concentrations of Calanquinone A. Western blot analysis revealed that the levels of phospho-ERK1/2, phospho-JNK, and phospho-p38 remained unchanged upon Calanquinone A treatment, indicating that this compound does not significantly impact MAPK pathway activation. Total protein levels of ERK1/2, JNK, and p38 were also unaffected.
